# Sustainable, healthy cities: protocol of a mixed methods evaluation of a cluster randomized controlled trial for *Aedes* control in Brazil using a community mobilization approach

**DOI:** 10.1186/s13063-019-3714-8

**Published:** 2020-02-14

**Authors:** Kate Zinszer, Andrea Caprara, Antonio Lima, Stéphanie Degroote, Monica Zahreddine, Kellyanne Abreu, Mabel Carabali, Katia Charland, Mayana Azevedo Dantas, José Wellington, Beatriz Parra, Florence Fournet, Emmanuel Bonnet, Denis Pérez, Emilie Robert, Christian Dagenais, Tarik Benmarhnia, Neil Andersson, Valéry Ridde

**Affiliations:** 1 School of Public Health, University of Montreal, Montréal, Québec Canada; 2 Québec Public Health Research Centre, Montréal, Canada; 3 Québec Population Health Research Network, Montréal, Canada; 4 Fortaleza Municipal Health Secretariat, Fortaleza, Brazil; 5 University of Fortaleza, Fortaleza, Brazil; 6 French Institute for Research on Sustainable Development, Paris, France; 7 McGill University, Montréal, Canada; 8 Universitario del Valle, Cali, Colombia; 9 Pedro Kourí Tropical Medicine Institute, Havana, Cuba; 10 SHERPA Research Centre, Montréal, Canada; 11 University of California, San Diego, USA; 12 Universidad Autonomy De Guerrero, Acapulco, Mexico

**Keywords:** Cluster randomized controlled trial, Dengue, Community-based intervention, Mixed methods, Community empowerment, Vector control, Brazil, *Aedes* mosquitos

## Abstract

**Background:**

Dengue is increasing in its global presence with an estimated 4 billion people at-risk of infection in at least 128 countries. Despite the promising results of EcoHealth and community mobilization approaches to *Aedes* reduction, more evidence of their efficacy on reducing dengue risk is needed. The principal research question is to determine if interventions based upon community mobilization reduce the risk of dengue virus infection among children 3 to 9 years old compared to usual dengue control practice in Fortaleza, Brazil.

**Methods:**

The present study will follow a pragmatic cluster randomized controlled trial (cRCT) design with randomization at the census tract level with equal allocation to the two arms. In each arm, there will be 34 clusters of 86 children between 3 to 9 years old for an expected total of 5848 children enrolled in the study, assuming a risk reduction of 29.5% based upon findings from a previous multi-site cRCT. The primary outcomes are rates of anti-dengue Immunoglobulin G (IgG) seroconversion and adult female *Aedes* density. The intervention is based upon a participatory health research approach, Socializing Evidence for Participatory Action (SEPA), where the research evidence is used to foster community engagement and ownership of the health issue and solution. Following allocation, intervention communities will develop and implement their own solutions that will likely include a wide variety of collective events and media approaches. Data collection activities over a period of 3 years include household visits for blood collection, household surveys, and entomological surveys; and qualitative activities including focus groups, in-depth interviews, and document analysis to evaluate the process, acceptability, fidelity, and sustainability of the intervention. Study participants will be aware of their assignment and all research staff will be blinded although the intervention assignment will likely be revealed to field staff through interaction with participants.

**Discussion:**

The results of our study will provide evidence on community mobilization as an intervention for dengue control. We anticipate that if community mobilization is effective in Fortaleza, the results of this study will help develop evidence-based vector control programs in Brazil, and also in other countries struggling with *Aedes*-transmitted diseases.

**Trial registration:**

ISRCTN66131315, registration date: 1 October 2018.

## Background

Dengue is increasing in its global presence with an estimated 4 billion people at-risk of infection in at least 128 countries [1–3]. Rising incidence and large-scale outbreaks are largely due to inadequate living conditions, naïve populations, rapid urbanization, global trade and population mobility, climate change, and the aggressive nature of the mosquito vectors *Aedes aegypti* and *Aedes albopictus* [4–6]. Poor quality housing and sanitation management, and high population density are key determinants of increased risk of infection at the population level [7, 8]. It is estimated that dengue, the fastest spreading mosquito-borne viral disease, infects 390 million people annually [4, 9] with a 30-fold increase in incidence during the past 50 years [10]. Dengue is highly endemic in Brazil with over 1.7 million cases reported for 2016 and 2017 [11, 12]. In Brazilian cities, the inconsistency in the supply of household piped water requires inhabitants to store clean water in water containers and water reservoirs creating ideal breeding sites for *Aede*s within and near the households [13].

There is no current treatment for dengue and vaccines are in different stages of commercial development, with one vaccine being licensed although its implementation is context specific [14–16]. Preventing or reducing dengue transmission primarily depends on controlling the mosquito vectors or interrupting human-vector contact. Many vector control options have been identified but the evidence of effectiveness is often conflicting or missing [17]. There is increasing resistance of the mosquitos to larvicides and insecticides, [18, 19] which have failed to contain *Aedes* expansion [20–22] or result in sustained reductions of mosquito populations [17]. In addition, there are important health concerns related to the chronic exposure of pesticides [23, 24]. A recent meta-review of systematic reviews stated that better quality studies of *Aedes* intervention studies are needed [17] with a systematic review of *Aedes* control randomized control trials demonstrated the promising results from community mobilization interventions [25].

With the cost and growing burden of dengue, it is an urgent priority to identify effective evidence-based control options [17, 21, 26] in endemic and at-risk regions. For dengue alone, worldwide estimates are as high as 39 billion USD per year on the costs of medical care, surveillance, vector control, and lost productivity [27]. Brazil is also prone to outbreaks of chikungunya and zika, which are transmitted by the same *Aedes* vectors. There were two waves of a chikungunya outbreak in 2016 and 2017 with a total of 445,274 cases [11, 12], and prior to the chikungunya outbreak, there was the introduction of zika. The zika outbreak began in 2015 and ended in 2017, which resulted in 231,566 cases, including 3014 cases of Congenital Zika Syndrome [28]. Given that immunity can be developed for chikungunya [29] and potentially for zika [30], and that the current number of cases for both diseases in Fortaleza is extremely low [31, 32], our study will focus on dengue infections.

A community mobilization trial, Camino Verde (IRSCTN27581154), based in Nicaragua and Mexico, demonstrated that community mobilization, as well as customization of activities to local contexts, were effective strategies for vector control and dengue reduction in a pesticide-free environment despite the differences between the two sites in socio-economic status, dengue prevalence, safety conditions, and community organization and support [7, 8]. Local pilot work conducted in Fortaleza included a cluster randomized controlled trial, which was conducted from 2012 to 2013 and demonstrated the effectiveness of an Ecohealth approach, including social participation, to reducing *Aedes* density [33]. Other local pilot work included a cluster randomized controlled which found that insecticide-treated curtains reduced seasonal dengue infections and *Aedes* mosquito density [34]. The design of our proposed trial is closely based on the Camino Verde study, which will occur in a different context in that it is highly urbanized and hyperendemic for dengue. We aim to evaluate the effectiveness of community mobilization in reducing the risk of dengue infections and mosquito infestation compared to usual dengue control practice in Fortaleza, Brazil.

## Objectives

The principal research question of this study is: does community mobilization reduce the risk of dengue virus (DENV) infection compared to usual dengue control practice in Fortaleza, Brazil? The specific objectives are 1) Measure the impact of the intervention on the risk of primary dengue infections using serological indicators in participating children; 2) Measure the impact of the intervention on human exposure to adult female *Aedes* in participating households; 3) Measure the impact of the intervention on self-reported dengue in participating households; 4) Explain the heterogeneity of the effect of community mobilization; 5) Analyze the acceptability of the intervention and the empowerment process of communities and individuals.

### Trial design

We will follow a parallel pragmatic cluster randomized controlled trial (cRCT) design with randomization at the census tract level with equal allocation to the two arms, to evaluate the effectiveness of community mobilization measured by the risk of primary DENV infection, self-reported dengue cases, and *Aedes* infestation rates. The quantitative baseline assessment will include a household questionnaire, entomological evaluation of households and high-risk communal areas, and blood samples (from finger pricks) from children aged 3–9 years. Following the baseline assessment, allocation will occur after which community mobilization activities will begin for a period of 24 months. Baseline community interviews and focus groups for the qualitative studies will be performed just after allocation.

This is an investigator-initiated trial that was registered with ISRCTN66131315 prior to enrollment. The trial protocol was developed according to the Standardized Protocol Items: Recommendations for Interventional Trials (SPIRIT) guidelines (Additional file 1) [35].

### Methods: participants, interventions, and outcomes

#### Study setting

The city of Fortaleza, capital of Ceará State, is situated on the Atlantic coast of north-eastern Brazil with a rainy season from January to May. Fortaleza is vulnerable to infestation of *Aedes aegypti* due to its tropical climate, high population density and rapid population growth (4.9%; from 2010 to 2014 [36]), and inadequate sanitary conditions [33, 37]. Irregular water supply leads people to store water in various containers such as water tanks, cisterns, barrels, drums, bowls, and pots. Fortaleza is particularly burdened by arboviruses. From 2016 to 2017, there were 35,159 cases of dengue, 79,486 cases of chikungunya, 1598 cases of zika, and 52 cases of congenital zika syndrome reported in Fortaleza [31, 32, 38]. It is important to note zika cases began to appear in large numbers in Fortaleza in 2015 although the compulsory notification did not begin until 2016. There are two currently circulating dengue serotypes in Fortaleza, DENV1 and DENV2 [39], with the public health officials anticipating the possibility of reintroduction of DENV3 within the next few years.

Standard dengue control practice activities of the Fortaleza Municipal Health Secretariat include periodic visits of houses and high-risk *Aedes* breeding grounds in communal areas (e.g., scrapyards, tire repair shops, etc.) by vector control agents for habitat destruction and application of the larvicide, Diflubenzuron, for containers that cannot be cleaned [40]. In Brazil, the use of temephos as a larvicide was discontinued in 2010 due to increased resistance and toxicity and the general population of Fortaleza do not have access to larvicides for domestic purposes. The Vector Control Department within the Fortaleza Municipal Health Secretariat is responsible for community education and mobilization actions in areas of higher transmission risk.

#### Eligibility criteria

During enrollment visits, all households in selected clusters with children from 3 to 9 years will be eligible and asked to participate in the trial [41]. Eligibility will be evaluated on three levels: cluster, household, and individual. The inclusion criteria will be: 1) any of the 3020 census tracts from the 2010 Census; 2) households permanently inhabited; 3) children aged 3 to 9 years. The exclusion criteria will be: 1) census tracts where interventions outside of vector control standard practices occurred within the last 5 years; 2) census tracts deemed to be too insecure for study personnel (determined based upon the opinion of the research team and stakeholders); 3) clusters with less than 230 households as previous work has shown that 230–240 households are required to obtain the needed sample size [8]; 4) abandoned or non-permanent households; 5) households with the intention to move outside of the household during the study period; 6) children with chronic disease or other health condition that preclude participation in the study; and 7) parents or guardians who are unable to give informed consent.

#### Intervention

Socializing evidence for participatory action (SEPA) is an approach to health promotion and community development based on the production and use of research evidence [7, 42]. Critical to SEPA is the socialization of research evidence to communities and leadership to foster engagement in finding solutions and local strategies to their own health problems. At allocation, community engagement will begin by presenting each intervention cluster with their baseline results through community meetings and household visits using community volunteers and facilitators from the research team. Volunteers from the communities will serve as organizers and educators trained by facilitators from the research team. Intervention design groups will be organized in each cluster with community members, including a subsample of people with opinions on vector control interventions, for the development and design of the customized vector control activities and the implementation process. Customized community activities will be developed during community meetings and based on pilot studies may include: i) neighbourhood clean-up campaigns; ii) distribution of intervention package to promote research activities and efforts to reduce vector breeding sites; iii) collaboration with community and municipal services to improve garbage collection and to cover collection bins (highly productive breeding sites of *Aedes* [43, 44]); iv) school visits; v) artistic demonstrations or sport competitions; vi) SMS communication. Community activities will be continually adapted post-implementation while ensuring a rigorous documentation of the process and evolution with the facilitators monitoring and documenting community activities. Intercommunity (or intercluster) visits will be organized for volunteers of each cluster to share experiences between communities and to strengthen the group dynamics and the collective preventive action. Community-to-community monitoring will allow volunteers to gather both quantitative and qualitative information and will be opportunity to provide peer-encouragement and training for intervention field staff and communities.

We expect a high level of compliance, above 95%, in the activities chosen by the community against *Aedes* based on experience from preliminary work [33, 45]. The content, coverage, frequency, and duration of community and household participation and activity in community mobilization will be monitored through the fidelity and process analyses [46, 47]. Our long-term relationships with local stakeholders and field staff will facilitate the engagement of households and communities and we will ensure engagement through i) using baseline survey results [7], ii) implementation of a local steering committee involving community representatives, and iii) involving community members in the evaluation process through a participatory evaluation and interpreting the heterogeneity assessment of impact.

The control and intervention arms will receive standard practices of vector control of the Fortaleza Municipal Health Secretariat during the trial including periodic visits of houses and high-risk *Aedes* breeding grounds in communal areas (e.g., scrapyards, tire repair shops, etc.) for habitat destruction and application of larvicide and insecticide when necessary, and health education activities, provide by vector control agents.

#### Outcomes

Primary outcomes are: rates of anti-dengue Immunoglobulin G (IgG) seroconversion (from negative to positive at follow-up) to evaluate the incidence of primary infections, and adult female *Aedes* density (number of adult female *Aedes* per household). Additional measurement of the antibodies waning rate or disappearance of detectable IgG antibodies (from positive at baseline to negative at follow-up), which may happen one to 2 years after primary dengue infections will determine the impact of the intervention on subsequent dengue secondary infections [48]. Secondary outcomes captured during household visits at baseline and follow-up are: i) anti-dengue IgG antibodies waning rates; ii) self-reported dengue during the most recent dengue season; iii) entomological indices: the container index (number of positive containers per household) and the premise condition index (how well the structure is maintain and yard), and, iv) knowledge, attitudes, and practices (KAP) for dengue control that will combined to create an index [49]. Secondary outcomes captured from the household survey, community focus groups, and in-depth individual interviews include: i) social acceptability of activities; ii) implementation fidelity and adaptability processes; iii) potential for sustainability, and iv) empowerment of individual and communities.

#### Participant timeline

**Fig. 1:**
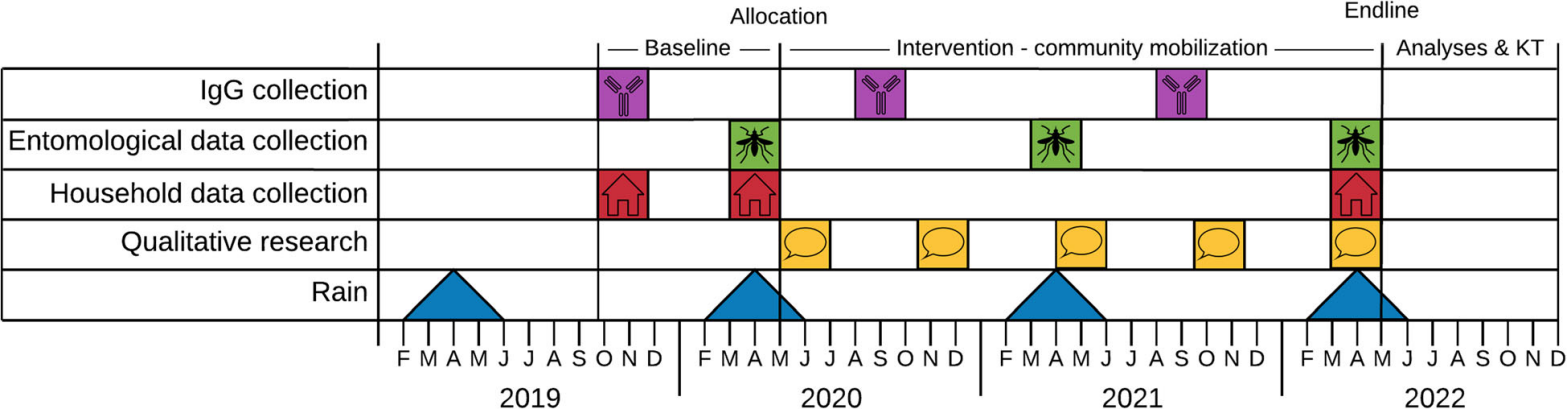
Summary of research activity timeline

**Fig. 2:**
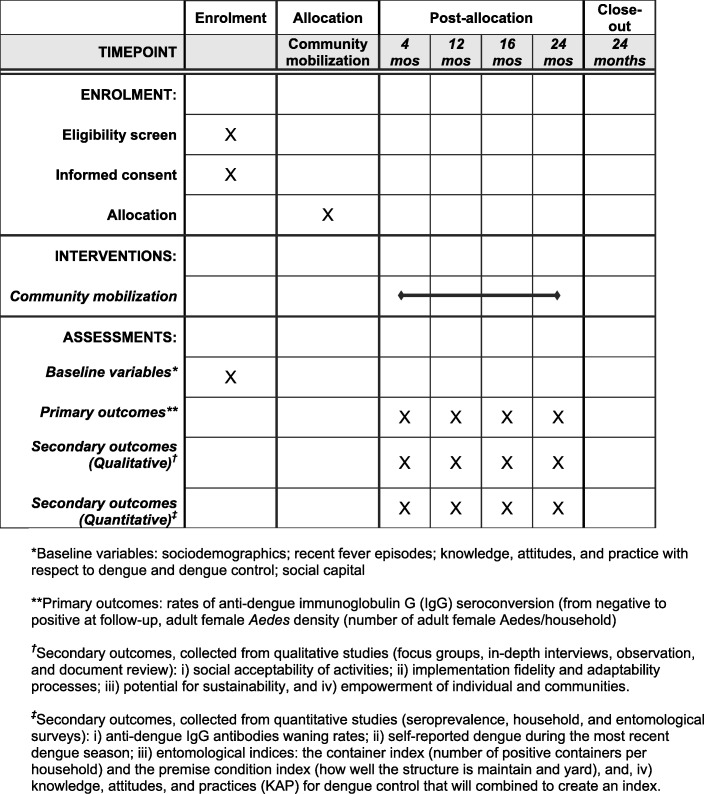
Overview of enrolment, intervention, and assessment as per the Standard Protocol Items Recommendations for Interventional Trials (SPIRIT) Statement

Household visits for the questionnaire will occur at dry season and rainy season baseline and at endline (Figs. 1 and 2). The seroprevalence baseline and follow-up surveys will occur yearly (for 3 years), 5 months after the rainy season peak to allow the maximum levels of IgG to be reached without residues of other immune reactions to cross-react [50]. A baseline entomological survey will occur in rainy season, the same period as the household questionnaire. Following baseline, entomological surveys will occur yearly during the rainy season. Community focus groups and interviews will occur twice a year, starting with the baseline assessment.

#### Sample size

Our pilot data, along with a research member’s (NA) RCT in Mexico and Nicaragua facilitated the sample size estimation. Our primary outcome of interest is risk of dengue seroconversion in 3 to 9 years old. The local pilot study’s intraclass correlation coefficient (ICC) was 0.08 across the 10 control clusters and the control group risk of seroconversion was approximately 0.30 (population 5–15 years old). The community mobilization RCT for dengue prevention by Andersson et al. [7] determined an ICC of 0.03 across 150 clusters for dengue seroconversion in children aged 3 to 9 and a relative risk reduction of 29.5% (95% CI 3.8 to 55.4). Using the approach of Reich et al. [51] for finding sample sizes to compare proportions between group, assuming a risk of seroconversion of 0.30 in the control group and 0.2115 in the intervention group (based on relative risk reduction of 29.5%) and an ICC of 0.07, for 80% power, a significance level of 0.05 and 60 individuals per cluster, 32 clusters are required in each arm. An additional two clusters per arm will be added, in the event that a cluster is removed from the study due to violence creating unsafe conditions for field staff [37, 52]. To have a sufficiently powered analysis in the case of a 30% attrition rate of participants, 60/0.7 = 86 individuals per cluster per arm are required. There will be a total of 5848 participants recruited for this study (86 individuals/cluster × 68 clusters).

#### Recruitment

Based on preliminary and exploratory studies [7, 8, 45, 53, 54], we observed high participation rates in seroprevalence surveys (93% to 100%) and the finger prick in children to collect blood samples was well accepted [45, 53–56]. During door-to-door enrolment visits the research teams will be assisted by community workers and volunteer facilitators to explain the aim and importance of the research to families. The starting point of household recruitment will be the centroid (as determined by QGIS) of each selected cluster with a radial pattern of household recruitment from the starting point, following the pattern of city blocks within the cluster.

Eligibility criteria of the household and individual will be verified. The goals of the study and all procedures will be detailed following a pre-established script, the consent forms will be explained, and we will allocate adequate time to answer any prospective participant questions. Immediately after enrolment and consent of participation, entomological data collection and/or blood collection, as well as questionnaires administration will occur.

### Assignment of interventions

#### Cluster selection

**Fig. 3:**
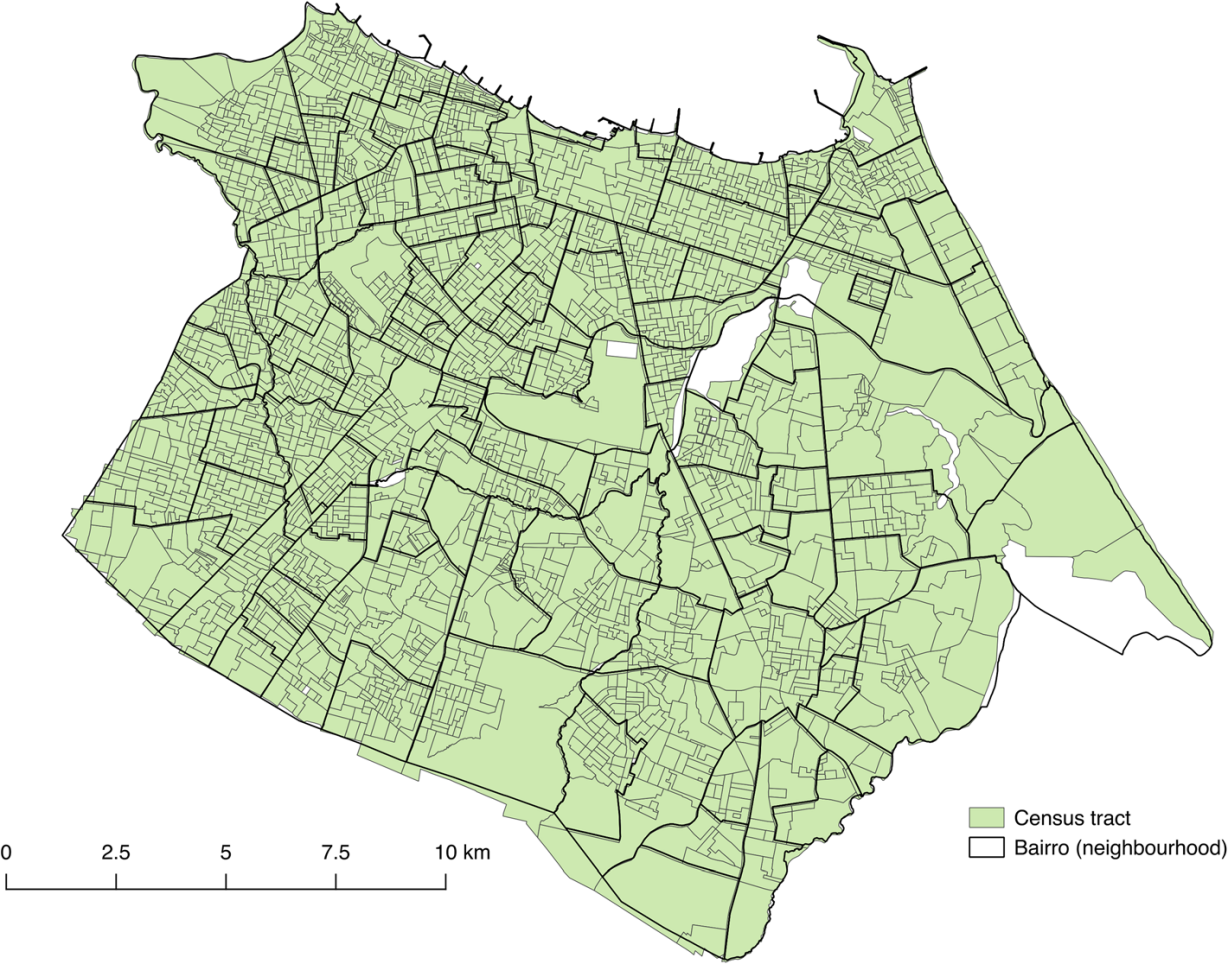
Map of Fortaleza

In Fortaleza, there are 3020 census tracts with approximately 230 households and 810 individuals in each census tract (Fig. 3).

Each census tract or cluster will be enumerated and the cluster eligibility criteria will be applied. Following the identification of eligible clusters, a proportionate stratified random sample of eligible clusters will be conducted. The stratification will be based on a dengue risk index calculated from the cumulative incidence of dengue notifications from 2016 to 2018 per neighbourhood or bairro, available from the Fortaleza Municipal Health Secretariat [57]. Census tracts within each bairro will be assigned to the same risk strata. Furthermore, a 400 m buffer for each cluster will be used to reduce contamination, creating a 800 m minimum distance between clusters. Clusters within that boundary area will be replaced by the next cluster, from a ranked list based upon stratification criteria [58].

#### Allocation

After both baseline collections, the rate of IgG seroconversion of each cluster will be calculated. Based on the overall distribution of seroconversion rate, there will be five risk strata created and each cluster from the baseline assessment will then be assigned to a risk stratum. A computer generated (block) randomization of each enumerated member of the strata will be assigned to either the intervention or control arm.

#### Blinding

Study participants will be aware of their assignment given that active participation in research design and activities will be encouraged for participants in the intervention group, whereas participation of those in the control group will not be solicited. Research staff involved in data collection will be blind to intervention assignment although intervention assignment may be revealed through interaction with the participants. During the analysis, all data will be anonymized, and research staff involved will be blinded to group assignment.

#### Contamination

Despite the protective measure of excluding clusters that are within 800 m from another cluster, there will likely be contamination through people interacting between the clusters at the individual or household level and through schools [8]. In addition, mosquitos from neighbouring households may influence rates in the intervention sites and children may get bitten by infected mosquitos outside of their intervention site. Both types of contamination will reduce the measured difference between control and intervention clusters although we will document the spread of intervention beyond the intervention clusters by monitoring any changes in behaviors within households and at the cluster level. Furthermore, we will collect school data from participating children including GPS coordinates for the schools and identify if the school is in a control or treatment area. This information will be included in the final analysis to estimate the impact of potential contamination on our findings.

### Data collection methods

#### Blood samples

Dried blood spot (DBS) samples will be collected at baseline and follow-up visits by trained nurse technicians, according to aseptic measures using disposable contact activated lancet and gloves. Finger prick will be used to facilitate the sampling procedure for children. A thin layer of lidocaine gel (topic local anesthetic) will be applied to ease the sting of the prick [59]. Expressed blood drops will be placed on filter paper [60–63]. All samples will be anonymized with study ID code upon collection and will be stored and labeled using barcoded stickers. Samples will be stored individually in a resealable plastic bag in a refrigerator between 4 °C and 1 °C until processing for analyses. DBS elution will be diluted for enzyme-linked immunoabsorbent assays (ELISA) using Panbio Dengue IgG Indirect ELISA kits for the detection of IgG antibodies to dengue antigen serotypes (1, 2, 3 and 4) in serum.

#### Entomological assessments

Each participating household will be inspected by entomological workers for all indoor and outdoor containers (tires, flower vases, water storage barrels, laundry wash basins, plastic tarps, puddles, and discarded containers such as bottles, cans, drums, metal pots, and plastic containers) including natural habitats such as tree holes and leaf axils [8]. Entomological surveys will also be conducted in elementary schools, identifying high-risk areas for breeding sites within each school as well as high-risk areas within the cluster (e.g., waste collecting bins, scrap yards, vacant lots, landfills). Schools and households will also have a premise condition index, which is based upon the house or school structure conditions (e.g., well-maintained) and yard conditions, if applicable, (e.g., well-maintained), which will range from 0 to 6 [64]. All containers will be classified according to size and use (routinely used or not in use), and presence (based on visual inspection) of larvae and/or pupae, which will be used to calculate the container index (number of positive containers per household). Adult mosquito collection will be conducted for each household using Prokopact aspirators, for a collection time of 1 h for both inside and outside of the home. Captured mosquitos will be stored on wet or dry ice in an insulating container and transported to the laboratory where all adult female *Aedes aegypti* and *Aedes albopictus* will be identified and stored. This will be used to calculate the number of adult female *Aedes* mosquitos per household. Ambient temperature and relative humidity will be recorded for each household during the entomological surveys using an iButton sensor.

#### Household survey

A questionnaire will be administered to a principal adult respondent by a trained interviewer during the entomological inspections and will include questions about: sociodemographics; recent dengue episodes; KAP [49] with respect to dengue and dengue control practices; social capital; acceptability [65] and participation in research activities. The wet season baseline will be shorter in length, focusing on recent dengue episodes and KAP.

#### Community focus groups and interviews

There will also be a series of focus groups convening representatives from six intervention communities as well as individual interviews, with a subsample of people with opinions on vector control interventions (principle of purposeful sampling, criterion-i) [66]. Intervention communities will be self-identified neighbourhoods that are not confined to the intervention clusters [7] although eight intervention clusters will be randomly chosen, according to a risk index which will be created from the baseline data using principal component analysis. The eight clusters will be selected to represent the range of heterogeneity of risk amongst the intervention clusters. Clusters will be used as a starting point in identifying intervention communities.

Focus groups, with at least 4 groups of 8 to 10 individuals each, will include representatives from each intervention community that will meet every 6 months. Individual interviews (25 to 30 people) will be conducted with key stakeholders from each intervention community every 6 months with data saturation taken into consideration. This information will be used for a series of analyses including process, social acceptability, fidelity, empowerment, and realist. All focus group and interviews will be carried by researchers in social sciences who will record, transcribe and analyse the data according to the framework analysis and realistic evaluation process [65, 67].

#### Document analysis

For the fidelity analysis [46, 68], a process documentation system will be created. Implementation communities will be identified post-allocation and the types of actors involved in each component or intervention activity. The critical points for assessment in the implementation process will be identified and self-report forms will be created based on specific descriptors of fidelity to the intervention (i.e. details of content, processes, frequency, duration, coverage) which will be completed by facilitators in each community. A purposive heterogeneous sample of the actors involved in the implementation will be trained in completing the forms systematically while implementing the interventions. The self-report forms will be collected every 6 months, coinciding with household data collection.

#### Data quality assurance

For serological analyses, the assay will be run with controls and cut off samples (quality control procedure) included within the ELISA kit and once the assay passes the quality control measures, unique samples will be tested. For individual with samples that seroconvert during follow-up, both samples from baseline and follow-up will be tested again in the same assay plate as controls for confirmatory testing. In addition, paired samples (baseline and follow-up surveys) will be used as procedures control to validate time consistency in the laboratory analyses. A random subsample of 10% of seroconverted samples for anti-DENV IgG will undergo PRNT for confirmation of infection, verify cross reaction with other arboviruses and serotyping. For the entomology samples, a chief lab entomologist will validate counting and classification by species for 1% of all samples.

Standard Operating Procedures (SOPs) will be adapted from previous studies and implemented for all procedures included in that trial. All research members and personnel will adhere to strict data quality assurance processes. At the beginning of trial preparation, a workshop will be held in Brazil for research members to discuss and develop guidelines and SOPs for research staff and field teams. The procedures’ stringent application will be supervised by the field coordinator. A two-day training session will be held for each field team to ensure good practice during data collection. The training will include different modules according to personnel needs: recruitment and interviews techniques, blood collection, entomological data collection, electronic data collection, confidentiality, and qualitative data collection. There will be field supervisors for blood collection, household surveys, entomological surveys, and qualitative data to ensure high quality data collection. Further quality assurance steps are described in the data monitoring section.

#### Approach for participant retention

Based on experience in pilot work ranging between 10% [7] to 20% [45] loss to follow-up with 2 visits, we have estimated a 30% rate of attrition in the current study, due to increased length of follow-up (4 follow-up visits). Participation and follow-up will be encouraged by offering at-home visits and the possibility of rescheduling visits when necessary. All data collection milestones will be scheduled in consultation with the local steering committee to determine the best possible time for the community. Advertisement will be made within the community prior the home visits for data collection to inform households of the upcoming visit of the research team. To compensate for losses due to attrition, decreased participation, and potential biological sample losses, we plan a 43% increase in recruitment.

### Data management

The data collected from the entomological and household assessments will be collected electronically on android phones, that have been programmed Open Data Kit (https://opendatakit.org) which is an open source software. This allows integrating questionnaires in a light application with the appropriate skip logic, value restrictions, and data quality checks before a survey can be finalized. Once a survey is complete, the data will be saved locally on the android phone and later with wifi access, will be synchronized via secure sockets layer (SSL) to a secure server with KoBoToolbox (https://www.kobotoolbox.org). Survey data, GPS coordinates of households and of communal *Aedes* breeding sites, and audio recordings from focus groups can be captured by Open Data Kit and then stored on KoboToolbox. This platform has several features including archiving data, descriptive statistics and visualizations of the data, basic maps, with several export formats possible. All other data, such as the laboratory results from the blood and entomological samples, temperature and humidity data, and the qualitative studies will be stored on a secure server with the French Institute for Research on Sustainable Development (IRD).

### Statistical methods

We will follow an intention-to-treat (ITT) approach. To estimate the potential impact of the intervention on the risk of dengue seroconversion, we propose a conditional (multilevel) modeling approach (i.e., using mixed effects models) [69]. To estimate the incidence risk ratio of seroconversion in the intervention group relative to the control group, a log-binomial model will be used which assumes a log link under the assumption of a binomial distribution [70]. Since the intervention effect of marginal and conditional models are only the same for the identity and log links [69], if convergence is an issue, a robust Poisson model with log link and robust variance will be used [70]. In addition to the fixed effect for the intervention, the log-binomial mixed model will have random effects for strata, measurement period (baseline, first follow-up, second follow-up), household, and census tract (cluster). The random effects for period will allow us to assess period by period differences in the risk of seroconversion. Also, as an alternative to the strata random effects, we will consider the baseline risk indicator (cumulative dengue incidence at the bairro level) that was used to stratify clusters. Additional baseline covariates may be considered for inclusion at the patient and household level (e.g., age, sex, socioeconomic status). We anticipate that the cluster sizes will remain relatively similar across all clusters but if losses due to attrition result in significant differences, we will then use inverse probability weights by cluster size in the analysis. For the primary outcomes of adult female *Aedes* density, we will use the same approach: a conditional (multilevel) modeling approach (i.e., using mixed effects models) with a Poisson distribution. For KAP and social capital, they will be examined as potential effect modifiers/confounders with each outcome. An index of KAP will be created using principal component analysis. Additionally, indicators on the community interventions captured from the fidelity analysis (e.g., content, coverage, frequency, and duration) may be included as fixed effects in the models to estimate the impact of intervention variability on the results.

Data will be analyzed descriptively immediately after the baseline survey to disseminate the results during the needs assessment. Following this, the data will be analyzed every 6 months for preliminary results with a global analysis at the end of follow-up. The qualitative research analyses obtained from community focus groups and interviews will occur after endline.

#### Additional and subgroup analyses

Given the potential for participants to have more than one dengue infection during the study period, the rate of dengue infection will be modelled using Poisson regression, while using random effects strata, measurement period (baseline, first follow-up, second follow-up), household, and census tract (cluster). Additional baseline covariates may be considered for inclusion at the patient and household level (e.g., age, sex, socioeconomic status).

We will assess any differences in the associations of intervention and risk of dengue seroconversion by age group (≤ 5 yrs. and ≥ 6 yrs) and by socio-economic status (SES). Separately for the age group and SES variables, we will conduct a Cochran Q test [71] to test for differential changes in risk across time points between the vulnerable and non-vulnerable subgroups. Then, we will extend the models to allow for differential effects by including interaction terms between the intervention variable and a dummy variable for the vulnerable subgroup of interest [72]. We will finally consider an Oaxaca-Blinder decomposition analyses [73] to decompose the treatment effect variable between subgroups and estimate the exact contribution of the intervention to changes in inequities occurring after the implementation of the intervention. These are secondary objectives and the study may not be sufficiently powered to detect important differences between these subgroups for one or both variables.

#### Spatial analyses

Spatial analyses will be carried out to study the extent to which cases of dengue are spatially differentiated, and assess predictors that could explain these differences. We will use local methods of aggregates detection in space. The spatial scan approach elaborated by Kulldorf [74] will be adapted for our purpose. Typically, the scan statistic is used to identify unusual clustering of cases, but we will capitalize on its properties to determine whether any observed spatial heterogeneity is due to a few distant spatial units or to larger geographic zones of varying risk [75]. Geographically weighted regressions [76] will enable understanding the relation between the outcomes of primary interest: IgG seroconversion and adult female *Aedes* density, while cluster analysis using Local Indicators of Spatial Association [77] will allow us to assess of the significant of spatially-concentrated cases. We will examine various environmental and population-level demographic spatial predictors that could explain the spatial heterogeneity of dengue transmission risk using a Bayesian hierarchical Poisson model.

#### Missing data

There will likely be in-migration and out-migration from the community and we will add new arrivals to the study (but will not follow those leaving the household clusters if they move outside of a study cluster). We do not expect there to be differential out-migration between intervention and control clusters. To decrease the potential selection bias resulting from informative censoring (differential attrition) we will 1) minimize loss to follow-up by performing home-based visits, and 2) at the analysis stage, make use of methodological techniques such as inverse probability of censoring weighting [78, 79], which enables estimation of the effect in the presence of informative censoring by weighting each participant according to their conditional probabilities of staying or leaving the study [80]. In addition, it has been shown that the participatory approach of community-based interventions enhances the participation and reduces the attrition in health interventions [7, 81, 82]. In any case, attrition will be reported and managed adequately to best standards for RCT validity [83]. We will analyse missing data using Amelia II [84] to impute values for missing data for the primary outcome (serological evidence of recent DENV infection). Estimates will reconcile data from ten imputed cross-sectional data sets in the R package Zelig [85] applied at multiple time points.

### Qualitative methods

#### Fidelity and process analysis

We will first incorporate feedback on the intervention’s theory of change from key stakeholders. To measure fidelity, programmed activities (defined with specific descriptors using questions formulated based on the theory of the intervention) and their actual implementation will be compared in terms of content, coverage, frequency, and duration [86]. The assessment of processes will focus on the internal dynamics of the interventions, the roles, perceptions and coping strategies of actors, adaptation to changes brought about by activities, implementation fidelity moderating factors (i.e. quality of delivery of the intervention, participants recruitment, responsiveness/acceptability and expectations, comprehensiveness of the intervention description, facilitations strategies and implementation context), barriers and facilitators of the implementation, any unintended effects, the evolution of programs and activities, and the mediating effect of the context [87–89]. Data will be collected from the self-report forms established for process documentation and from in-depth interviews with a purposive heterogeneous sample of implementation from key stakeholders. We will assess the participation process using a participation framework and indicators [90, 91]. The fidelity data will be analysed with the Perez et al. [46] framework and qualitative data will be analysed using an inductive approach [92]. Component analysis will be also conducted by using implementation fidelity data and outcomes performance from different implementation units of the intervention.

#### Social acceptability analysis

This analysis will be performed on two levels with a mixed methods approach using the theoretical framework proposed by Sekhon et al. [65]. The acceptability of stakeholders involved in designing and implementing the interventions will be assessed in the development and adaptive process of the activities. The qualitative data will be collected through the in-depth interviews planned for assessed fidelity moderators mentioned above. Acceptability will also be assessed with quantitative data at household level as part of the household surveys. The qualitative data will be analysed using a framework analysis approach [92, 93] and the household survey data will be analysed using a generalized estimating equation approach for proportional odds logistic regression model with repeated measures [94].

#### Realist analysis

Realist evaluation (RE) [67] is an innovative approach to understand complex social interventions such as community mobilization. Using generative causation, RE aims to highlight cause-and-effect tendencies in the occurrence of an outcome in a specific context by determining the reasoning and reactions of agent(s) [95]. RE will focus on: i) participation of stakeholders; ii) adaptation of interventions; and iii) education and awareness of target populations. An embedded multiple-case study design will be used [96], with the three entities of analysis. These will include participants, neighbourhoods, and households. First, we will develop concrete theories underlying the potential effects of specific programme mechanisms through a workshop involving key stakeholders. Second, the research team will generate hypotheses from the theoretical literature to explain the process by which the intervention may produce the outcomes in certain contexts. Third, the hypotheses will be tested on empirical data, in order to clarify interactions between contexts, mechanisms and outcomes. Empirical data will be provided by the same focus group and individual interviews with communities in addition of individual interviews with key stakeholders involved in the intervention implementation, as well as the results of the KAP questions from the household survey.

#### Sustainability analysis

A planned and regular documentation of the project using the Template for Intervention Description and Replication for Population and Health Interventions [97], as well as in-depth interview with key players of the intervention, and cost data will help us evaluate the potential for sustainability of the intervention. We will use the five programmatic characteristics (leadership, capacity, interactions (notions of integration), flexibility/adaptability and performance) proposed by Shigayeva and Coker (2015) [98] to perform this analysis. The qualitative data will be analysed using a framework analysis approach [93].

### Methods: monitoring

#### Data monitoring

An external data monitoring committee will not be appointed given the low-risk to participant safety, although there will be an internal Data & Technology committee that will manage the data monitoring and evaluation for data quality assurance. Data monitoring will occur in real-time to examine response rates and numbers of interviews, missingness and other data quality issues, location match checking, and to identify problematic questions or interviewers. Data will be analyzed immediately after the baseline survey to disseminate the results during the needs assessment. Following this, the quantitative and qualitative data will be analyzed every 6 months following data collection for preliminary results with a global analysis at the end of follow-up. The results of the dengue incidence testing in study participants will be reported to Epidemiology Surveillance Division of the Fortaleza Municipal Health Secretariat.

#### Harms

The nurses who will be conducted the finger pricks, will be encouraged to report any potential adverse events (e.g., haemolysis, severe pain) to the investigators, who will report these findings to the governing institutional review boards [99].

### Ethics and dissemination

#### Consent

Consent forms will be presented and the study explained to household principal respondents to obtain the authorization of the household participation, using a standard script. They will explain that participants may decline to answer any questions and may terminate the interview at any time, which will proceed if the respondents have provided their consent. An additional consent form will be presented and explained to the parent or legal guardian of the eligible child, to obtain blood samples via finger pricks, with the assurance that the results will be returned to them. The study and the procedure for blood collection will also be explained to the child in an age adapted language, who will be asked for study participation acceptance using an assent form, which may include the use of visual aids.

#### Confidentiality

Maintaining confidentiality of participants and communities will be central to the training of fieldworkers and data operators. Only group findings will be reported with no names or personal identifiers recorded next to individual responses. All surveys and laboratory specimens will be identified by a coded ID, which will be used to link households and children participants to their information. All records that contain names or other personal identifiers will be stored separately and will have restrict access: paper informed consent will be stored under key at the Universidade Estadual do Ceará, and coded IDs file in a password protected database. These data will be kept for 7 years after the end of the project (December 2029). Anonymized data will be safely stored in a backup platform of the Institut de recherche pour le development (IRD), France, and protected by secure access. In focus groups, no names of focus group participants will be recorded, and reports of focus group findings will not identify individual communities. All principal investigators will be given access to the cleaned, final datasets.

#### Dissemination

Knowledge translation is integrated throughout our research study [100]. We will share study results with the participants, including after the baseline assessment, through community meetings and the study website. Parents or guardians of children participants will be informed of the status of their child’s test results via a call from a study nurse. Wide dissemination of our findings will occur with the Fortaleza Municipal Health Secretariat and the Brazilian National Institute of Health through policy briefs and deliberative workshops. Policy briefs will use infographic material and written in plain language to summarize the key elements of the study and its results. They will also propose clear recommendations for action based on the results produced. Deliberative dialogue [101, 102] is a workshop that allows research evidence to be considered together with the views, experiences and tacit knowledge of those who will be involved in, or affected by, future decisions about high priority issues [103]. Deliberative dialogues have been documented as improving the acquisition of new knowledge, the intention to use the research evidence, and have led to concrete actions aimed at implementing recommendations emerging from the dialogue [104]. We will also disseminate our results to other endemic countries through our policy briefs as well as through international and regional *Aedes* and arboviruses networks, notably AEDES Network, DENTARGET, WHO/TDR, and PAHO. We will disseminate to the broader scientific community through open access publications and presentations at national and international conferences, and authorship will be determined according to our internal authorship guidelines. We also make aggregate data of the study’s findings publicly available, after publication, through the study website.

## Discussion

Community mobilization is a promising approach to dengue control in that it inherently involves strong community engagement and participation as well as local customization of interventions [105]. There is an important need to determine the effectiveness of community mobilization in different contexts through high quality studies with sufficient follow-up periods including process and fidelity evaluations. Our study also contains several innovative aspects including embedded qualitative research that will determine the potential sustainability of community mobilization in Fortaleza and also the evolution of community engagement and intervention development and how this variation influences the intervention impact. It is expected that the results of this work provide further evidence on community mobilization as an intervention for dengue control in endemic countries. We anticipate that if the intervention of community mobilization is effective in Fortaleza, the results of this study will help develop evidence-based vector control programs in Brazil and in other countries struggling with *Aedes*-transmitted diseases.

## Trial status

Recruitment of participants began on 15 November 2019 and will be completed by 30 June 2020. The protocol version number is COESA-FORTALEZA-2019, dated 15 December 2019.

## Supplementary information

**Additional file 1.** SPIRIT 2013 Checklist: Recommended items to address in a clinical trial protocol and related documents*.

## Data Availability

Aggregate data generated from this data will be made available through the study website.

## Abbreviations

CI: Confidence interval
cRCT: Cluster randomized controlled trial
DBS: Dried blood spot
DENV: Dengue virus
ELISA: Enzyme-linked immunosorbent assays
GPS: Global positioning system
ICC: Intraclass correlation coefficient
IgG: Immunoglobulin G
IRD: French Institute for Research on Sustainable Development
ITT: Intention-to-treat
KAP: Knowledge, attitudes, and practices
PAHO: Pan American Health Organization
PRNT: Plaque reduction neutralization test
RE: Realist evaluation
SES: Socio-economic status
SMS: Short messaging service
SOPs: Standard operating procedures
SSL: Secure sockets layer
TDR: Special Programme for Research and Training in Tropical Diseases
WHO: World Health Organization
